# Salicylic acid-related cotton (*Gossypium arboreum*) ribosomal protein GaRPL18 contributes to resistance to *Verticillium dahliae*

**DOI:** 10.1186/s12870-017-1007-5

**Published:** 2017-03-03

**Authors:** Qian Gong, Zhaoen Yang, Xiaoqian Wang, Hamama Islam Butt, Eryong Chen, Shoupu He, Chaojun Zhang, Xueyan Zhang, Fuguang Li

**Affiliations:** State Key Laboratory of Cotton Biology, Institute of Cotton Research, Chinese Academy of Agricultural Sciences, Anyang, 455000 China

**Keywords:** Cotton, *Verticillium* wilt, Resistance gene, Ribosomal protein, *GaRPL18*, Salicylic acid

## Abstract

**Background:**

*Verticillium dahliae* is a phytopathogenic fungal pathogen that causes vascular wilt diseases responsible for considerable decreases in cotton yields. The complex mechanism underlying cotton resistance to *Verticillium* wilt remains uncharacterized. Identifying an endogenous resistance gene may be useful for controlling this disease.

**Results:**

We cloned the *ribosomal protein L18* (*GaRPL18)* gene, which mediates resistance to *Verticillium* wilt, from a wilt-resistant cotton species (*Gossypium arboreum*). We then characterized the function of this gene in cotton and *Arabidopsis thaliana* plants. *GaRPL18* encodes a 60S ribosomal protein subunit important for intracellular protein biosynthesis. However, previous studies revealed that some ribosomal proteins are also inhibitory toward oncogenesis and congenital diseases in humans and play a role in plant disease defense. Here, we observed that *V. dahliae* infections induce *GaRPL18* expression. Furthermore, we determined that the *GaRPL18* expression pattern is consistent with the disease resistance level of different cotton varieties. *GaRPL18* expression is upregulated by salicylic acid (SA) treatments, suggesting the involvement of *GaRPL18* in the SA signal transduction pathway. Virus-induced gene silencing technology was used to determine whether the *GaRPL18* expression level influences cotton disease resistance*.* Wilt-resistant cotton species in which *GaRPL18* was silenced became more susceptible to *V. dahliae* than the control plants because of a significant decrease in the abundance of immune-related molecules. We also transformed *A. thaliana* ecotype Columbia (Col-0) plants with *GaRPL18* according to the floral dip method*.* The plants overexpressing *GaRPL18* were more resistant to *V. dahliae* infections than the wild-type Col-0 plants*.* The enhanced resistance of transgenic *A. thaliana* plants to *V. dahliae* is likely mediated by the SA pathway.

**Conclusion:**

Our findings provide new insights into the role of *GaRPL18*, indicating that it plays a crucial role in resistance to cotton “cancer”, also known as *Verticillium* wilt, mainly regulated by an SA-related signaling pathway mechanism.

**Electronic supplementary material:**

The online version of this article (doi:10.1186/s12870-017-1007-5) contains supplementary material, which is available to authorized users.

## Background

*Verticillium dahliae* Kleb. is a destructive phytopathogenic fungus that causes wilt diseases on more than 400 plant species, including cotton (*Gossypium arboreum*) [1, 2]. *Verticillium dahliae* infects cotton by penetrating the roots. It then spreads across the root cortex and invades the xylem vessels where it forms the conidia responsible for the colonization of vascular tissues and functional impairment. This results in several symptoms, including wilting, discoloration, necrosis, and defoliation [3–6]. Cotton fiber quality and annual yields decrease as a result of *Verticillium* wilt induced by *V. dahliae*, and a severe outbreak can lead to yield losses of more than 30% [7, 8]. In China, more than 40% of the cotton-growing area is threatened by *Verticillium* wilt, potentially causing considerable decreases in cotton production and serious economic losses each year. Furthermore, the fungus can survive for long periods in the soil even without a host, making *Verticillium* wilt difficult to control using practical and effective chemical treatments [9, 10]. Numerous methods are used to reduce the incidence of *Verticillium* wilt, such as the application of tillage, soil solarization, soil amendments, and biological controls. However, these are not always efficient or effective [11, 12]. Soil fumigation, which is by far the most effective treatment for inhibiting the propagation of *Verticillium* species, is costly and can have lethal effects on human health and the environment [7, 13]. The identification and isolation of disease-responsive candidate genes, along with the development of disease-resistant transgenic cotton cultivars, are essential for managing *Verticillium* wilt [14–16].

The ribosomal protein (RP) has complex structures that differ in prokaryotes and eukaryotes. The eukaryotic ribosome is composed of two unequal subunits (60S and 40S), four ribosomal RNAs (rRNAs), and 82 different RPs. The small ribosomal subunit is composed of a single 18S rRNA and approximately 33 proteins, while the large subunit comprises 28S/25S, 5.8S, and 5S rRNAs, as well as approximately 49 proteins [17–19]. The ribosome is a highly conserved protein that is essential for cellular activities. Although its main function is to synthesize proteins, recent in-depth studies have revealed that it is also important for cell growth, division, and development, and gene regulation [20–22]. Recently, a study has shown that overexpression of the N-terminal 99 amino acids of ribosomal protein L3 confers resistance to pokeweed antiviral protein and the *Fusarium* mycotoxin deoxynivalenol in tobacco [23]. Another study has shown that ribosomal protein L12 and ribosomal protein L19 are important in nonhost disease resistance in *N. benthamiana* and *A. thaliana*. In addition, these genes also play a minor role in basal resistance against virulent pathogens [24]. In particular, a recent study examining ribosomal protein S14 (RPS14) and cancer concluded that this protein can specifically interact with murine double minute 2 (MDM2) to inhibit the degradation of p53 by MDM2 ubiquitin, thereby promoting p53 activity. In gastric and colorectal cancer cells the cell cycle is arrested and tumor cell growth is inhibited in the presence of abundant RPS14 [25]. Another study revealed that ribosomal protein L4 can also regulate the MDM2–p53 loop to regulate p53 activity [26]. Although these studies suggest that RPs are important for disease resistance, they did not include cotton species. Therefore, a more thorough characterization of the function of the cotton RP may be useful for the breeding of *Verticillium* wilt-resistant cotton varieties.

Under natural conditions, plants frequently encounter diverse potential pathogens. Plants are constantly evolving to cope with these biotic stresses. For example, plants have evolved an immune system that includes constitutive and inducible defense systems that offer protection from potentially dangerous pathogens [27, 28]. Plants also produce several endogenous signaling molecules that help regulate plant defense responses, including jasmonic acid (JA), salicylic acid (SA), and ethylene (ET), all of which are involved in complex signal transduction networks. These biochemical molecules function cooperatively or antagonistically to increase plant resistance to different pathogens [29–32]. Our study revealed that cotton *ribosomal protein L18* (*GaRPL18*) expression levels can be upregulated by accumulated SA, suggesting that RPs can mediate cotton resistance to *Verticillium* wilt through the SA signaling pathway. While SA is crucial for plant defenses and acquired systemic resistance, it is predominantly involved in the former [33, 34]. Increased SA levels in plant pathogen-challenged tissues and applications of exogenous SA induce the expression of *pathogenesis-related* (*PR*) genes, thereby enhancing resistance to invading pathogens [35, 36]. The activation of plant immune responses is also associated with increases in the production of reactive oxygen intermediates and nitric oxide (NO) levels [37]. While the signal transduction networks underlying all plant response mechanisms are complex, crosstalk between the different signaling molecules and networks provides plants with a powerful means of regulating immune responses [38, 39].

In this study, we focused on determining whether *GaRPL18* is important for cotton resistance to *Verticillium* wilt caused by *V. dahliae*. Our objective was also to identify the signaling pathway associated with cotton defense responses. To verify the expression of *GaRPL18*, we harvested *G. arboreum* ‘HuNanChangDeTieZiMian’ samples at different time points after treatments with *V. dahliae*, methyl jasmonate (MeJA), SA, or ET. We observed that the *GaRPL18* expression level increased significantly following *V. dahliae* and SA treatments. Moreover, we used virus-induced gene silencing (VIGS) technology and transgenic *Arabidopsis thaliana* lines overexpressing *GaRPL18* to functionally characterize *GaRPL18* in cotton. Complementary physiology and molecular experiments confirmed that *GaRPL18* significantly contributes to cotton resistance against the fungal wilt pathogen *V. dahliae* via a mechanism related to the SA signaling pathway. Our findings provide insights into the molecular features and functions of a cotton RP gene related to increased resistance to *Verticillium* wilt*.*

## Methods

### Plant sources and growth conditions

Seeds of *G. arboreum* ‘HuNanChangDeTieZiMian’ (resistant) and ‘NaShangQuXiaoHua’ (susceptible) were obtained from the Institute of Cotton Research of the Chinese Academy of Agricultural Sciences. The *GaRPL18* overexpression vector (i.e., *35S::GaRPL18*) was inserted into wild-type (WT) *A. thaliana* Columbia ecotype (Col-0) plant. The transgenic plants transformed with *35S::GaRPL18* were grown in a greenhouse at 22 °C and 60% relative humidity under a 16-h light/8-h dark photoperiod. The seeds from different *G. arboreum* cultivars were incubated in another greenhouse at 25 °C and 80% relative humidity under a 16-h light/8-h dark photoperiod.

### Culturing of *Verticillium dahliae* and inoculation of plants

An antagonistic defoliating *Verticillium dahliae* isolate (Vd07038) was grown on potato dextrose agar medium at 25 °C for 6 days. Colonies were then cultured in Czapek’s medium [3% (w/v) sucrose, 0.2% (w/v) NaNO_3_, 0.05% (w/v) MgSO_4_ · 7H_2_O, 0.05% (w/v) KCl, 0.002% (w/v) FeSO_4_ · 7H_2_O, and 0.131% (w/v) KH_2_PO_4_] for 5–7 days at 25 °C with shaking (150 rpm). For *V. dahliae* treatments, 10 ml conidial suspensions (10^7^ conidia/ml in sterile distilled water) were applied to the bottom of pots containing seedlings. Similarly, *A. thaliana* plants were grown for 20 days before a 10-ml conidial suspension was injected into the soil using a sterile needle. Control plants were inoculated with an equal volume of sterile distilled water. For in vitro treatments, seedlings were inoculated with *V. dahliae*, and the extent of stunting was determined using a previously described method [40]. Seedlings were inoculated with a 2-μl conidial suspension (5 × 10^3^ conidia/ml) 2 weeks after germinating.

### cDNA cloning and construction of the *GaRPL18* overexpression construct

Total RNA was extracted from *V. dahliae*-resistant *G. arboreum ‘*HuNanChangDeTieZiMian’ plants using the RNAprep Pure Plant Kit (Tiangen, Beijing, China). The purified RNA was used as a template to prepare cDNA with the PrimeScript™ II 1st Strand cDNA Synthesis Kit (Takara, Dalian, China). The 450-bp full-length *GaRPL18* coding sequence with *Xba*I and *Asc*I linkers was cloned using the primers GaRPL18-F and GaRPL18-R (Table 1). For overexpression studies, the *35S::GaRPL18* vector was constructed by digesting the *GaRPL18* coding sequence with *Xba*I and *Asc*I (BioLabs). The digested sequence was then inserted into a modified pCAMBIA3300 (Cambia) plant binary vector containing the *glufosinate* (*Basta*) resistance gene. This vector was used to transform *Agrobacterium tumefaciens* strain GV3101 using a freeze–thaw method.

**Table 1:** primers used in the research

Primer name	Forward and reverse primers(5’-3’)
GaRPL18-F	TCTAGAATGAAGCTTTGGGCCACCAA
GaRPL18-R	GGCGCGCCCATAAACAAGTTGGGTTT
VGaRPL18-F	ACTAGTATGAAGCTTTGGGCCACCAA
VGaRPL18-R	GGCGCGCCCATAAACAAGTTGGGTTT
QGaRPL18-F	AATGAAGTCCGTGCCAAATCCAAG
QGaRPL18-R	CGGAGCCAAATGCCGTAGTTCTTTC
Gahistone3-F	AAGACTGATTTGCGTTTCCA
Gahistone3-R	GCGCAAAGGTTGGTGTCTTC
AtUBQ10-F	AACTTTGGTTTGTGTTTTGG
AtUBQ10-R	TCGACTTGTCATTAGAAAGAAAGAGATAA
V-QPCR-F	AACAACAGTCCGATGGATAATTC
V-QPCR-R	GTACCGGGCTCGAGATCG
PAL-F	TGGTGGCTGAGTTTAGGAAA
PAL-R	TGAGTGAGGCAATGTGTGA
4CL-F	ATTCAAAAGGGAGATGCC
4CL-R	GAGAAGGGCAAAGCAACA
Basic chitinase-F	CTTAGCCCAAACTTCCCA
Basic chitinase-R	TACATTGAGTCCACCGAGAC
*β*-1, 3-glucanase-F	CACAGGTGCTGAAGTTGGT
*β*-1, 3-glucanase-R	CGATGGAGGGAAAGATGA
Cadinene synthase-F	TAACAACAATGATGCCGAGAA
Cadinene synthase-R	ATGGTCCAAAGATGCTACTGC
AtORA59-F	TCATTTGACCAATCCTTCCTTT
AtORA59-R	CCGTTTCCRCACRCCTCTGTAT
AtPDFl.2-F	ACCCTTATCTTCGCTGCTCTTG
AtPDFl.2-R	ATGTCCCACTTGGCTTCTCG
AtVSP2-F	CTTTCACTTCTCTTGCTCTTGGC
AtVSP2-R	GCAGTTGGCGTAGTTGATGGA
AtNPRl-F	GGCTTGCGGAGAAGACGAC
AtNPRl-R	ACGACGATGAGAGAGTTTACGG
AtPRl-F	GCTACGCAGAACAACTAAGAGGC
AtPRl-R	CCAGACAAGTCACCGCTACC
AtPR3-F	GAGACACCGCCACGAGGAA
AtPR3-R	TTGCTTGAAACAGTAGCCCCAT
AtAC02-F	TGTTCCTCCTCTCAACCACTC
AtAC02-R	CCGACATCCTGTTTCCTTCT
AtEIN3-F	TCAAGGCTTTGTTTATGGGATTA
AtEIN3-R	GCAAGGTATGAGGAGTCGGTC
AtERFl-F	GAGAATGACCAATAAGAAGACGAA
AtERFl-R	CTCCCAAATCCTCAAAGACAAC

*F* Forward primer, *R* Reverse primer

### Bioinformatics analysis

We used the National Center for Biotechnology Information online BLAST tool to analyze the *GaRPL18* sequence (https://blast.ncbi.nlm.nih.gov/Blast.cgi). The Gene Structure Display Server 2.0 was then used to analyze gene structures. We also used the ExPASy online tool (http://web.expasy.org/compute_pi/) to predict the isoelectric point and molecular weight. An image of the 3D structure was developed with the PyMOL program (http://www.pymol.org/).

### Generation of the virus-induced gene silencing construct and pathogen inoculation

We used *Cotton leaf crumple virus* (CLCrV)-based vectors (i.e., pCLCrVA and pCLCrVB) for VIGS, with CLCrV:CHLI (encoding magnesium chelatase subunit I) as a positive control [41, 42]. The *GaRPL18* fragment was amplified by polymerase chain reaction (PCR) using *‘*HuNanChangDeTieZiMian’ cDNA and the VGaRPL18-F/VGaRPL18-R primers (Table 1). The PCR product was digested with *Spe*I and *Asc*I (BioLabs) and inserted into the pCLCrVA vector. The constructs (i.e., pCLCrVA-*GaRPL18*, pCLCrVA, and pCLCrVB) were then used to transform *A. tumefaciens* strain GV3101. The cotyledons of 7-day-old *Verticillium* wilt-resistant cotton seedlings were then injected with equal amounts of CLCrV vectors. After a 24-h incubation in darkness, the cotton seedlings were transferred to the greenhouse and inoculated with *V. dahliae,*10 days after the vector infiltration.

### *Arabidopsis thaliana* transformation and molecular analysis

*Agrobacterium tumefaciens* strain GV3101 containing the *GaRPL18* overexpression vector was used to transform *Arabidopsis* Col-0 via the floral dip method [43]. The T_0_ transgenic seeds were then spread evenly over soil in a pot. After 1 week, seedlings were sprayed with 0.1% Basta to select positive transformants. The false-positive seedlings turned yellow before dying. Transgenic seeds of the T_1_ generation were selected on plates of Murashige and Skoog (MS) medium containing 0.1% Basta. After a few days, lines with segregation ratios of approximately 3:1 (i.e., Basta resistant: Basta sensitive) were used to generate T_2_ lines. The transgenic seeds of the T_2_ generation were also selected on MS medium containing Basta to identify T_3_ homozygous lines. The T_3_ lines with the transgene and the correct segregation ratio were detected based on quantitative reverse transcription (qRT)-PCR analysis of *GaRPL18* expression. Only stable homozygous T_4_ lines exhibiting high *GaRPL18* expression levels were chosen for further functional analyses.

### Quantitative reverse transcription polymerase chain reaction

We extracted total RNA from the roots and leaves of cotton plants as well as from the leaves of *GaRPL18*-overexpressing *A. thaliana* and WT plants using the RNAprep Pure Plant Kit. The RNA was used to prepare cDNA with the PrimeScript™ RT Reagent Kit with gDNA Eraser (Perfect Real Time; Takara). The *Gahistone3* (Cotton_A_11188) and *ubiquitin10* (accession: At4g05320) genes were used as internal controls for cotton and *A. thaliana*, respectively. We designed all qRT-PCR primers with the Primer Premier 6.0 program (Table 1). Diluted cDNA was used as the template for the qRT-PCR, which was conducted with SYBR^®^ Premix Ex Taq™ (Tli RNaseH Plus; Takara) and an ABI 7900 qRT-PCR System (Applied Biosystems, CA, USA). The three-step method involved the following PCR conditions: 40 cycles of 95 °C for 30 s, 95 °C for 5 s, and 60 °C for 30 s. We analyzed the dissociation curves for each reaction and used the 2^−ΔΔCT^ method [44] to calculate the expression level of each target gene. All reactions were conducted with at least three biological replicates.

### Quantification of *Verticillium dahliae* colonization

We used a previously described qRT-PCR approach to detect and quantify *V. dahliae* colonization. The qRT-PCR analysis with the V-QPCR-F and V-QPCR-R primer pair (Table 1) was completed as previously described [45].

### Methyl jasmonate, salicylic acid, and ethylene treatments

Plants were treated with 1 mM MeJA, SA, or ET solutions. Cotton or *A. thaliana* seedlings were grown in pots incubated in a greenhouse. They were sprayed with different solutions at the foliar stage. Control plants were treated with water at the same pH.

### Measurements of free salicylic acid, nitric oxide, H_2_O_2_, and catalase levels

The abundance of the immune system-related molecules SA, NO, H_2_O_2_, and catalase (CAT) was monitored using different methods. The free SA content was determined via the Rigol L3000 high performance liquid chromatography system (Beijing, China) as previously described [46]. We ground leaf samples in liquid nitrogen for subsequent measurements of NO, H_2_O_2_, and CAT levels using a Quantitative Assay Kit (Nanjing Jiancheng, Beijing, China).

### Cell death assay

Plant cell death was visualized with trypan blue staining as previously described with several modifications [47]. Leaves were soaked in trypan blue dye (1 g phenol, 1 mg trypan blue, 1 ml lactic acid, and 1 ml glycerol dissolved in 1 ml sterile distilled water) and then stained by boiling. After cooling to room temperature, samples were decolorized with a chloral hydrate solution (2.5 g/ml).

### *Verticillium dahliae* recovery assay

To determine the effects of a *V. dahliae* infection on cotton and *A. thaliana* plants, we analyzed stem fragments from the first stem node. The cotton and *A. thaliana* stems were 4.5 cm and 3 cm long, respectively. The stems were cleaned according to a previously described method [48], and then sliced into six parts. The stem fragments were placed on potato dextrose agar in plates, which were incubated at 25 °C. Plant susceptibility to infection was defined according to the number of stem sections from which the fungus grew.

### Analysis of the disease index, stunting, and chlorosis

For cotton plants, the disease index (DI) was calculated according to the following formula: DI = [(∑disease grades × number of infected plants)/(total checked plants × 4)] × 100%. Seedlings were classified into five grades (i.e., grade 0, 1, 2, 3, and 4) according to the symptoms on the cotyledons and true leaves [49, 50]. The disease severity for *A. thaliana* plants was graded on a 0–5 scale, and the DI was calculated with the following formula as previously described [40]: DI = [(∑disease grades × number of infected plants)/(total checked plants × 5)] × 100%. The DI represents a comprehensive and objective measure of plant health, with high DI values corresponding to serious infections. The extent of stunting was rated on a 0–3 scale (0 = no stunting; 1 = moderate reduction in leaf area; 2 = considerable decrease in leaf area; and 3 = considerable decrease in leaf area, leaf number, and stem length). Leaf chlorosis was rated on a 0–4 scale (0 = no symptoms; 1 = up to 25% chlorotic leaves; 2 = up to 50% chlorotic leaves; 3 = up to 75% chlorotic leaves; and 4 = up to 100% chlorotic leaves) [40].

## Results

### Analysis of *GaRPL18* structure and expression patterns in cotton plants treated with *Verticillium dahliae* or hormones

**Fig. 1:**
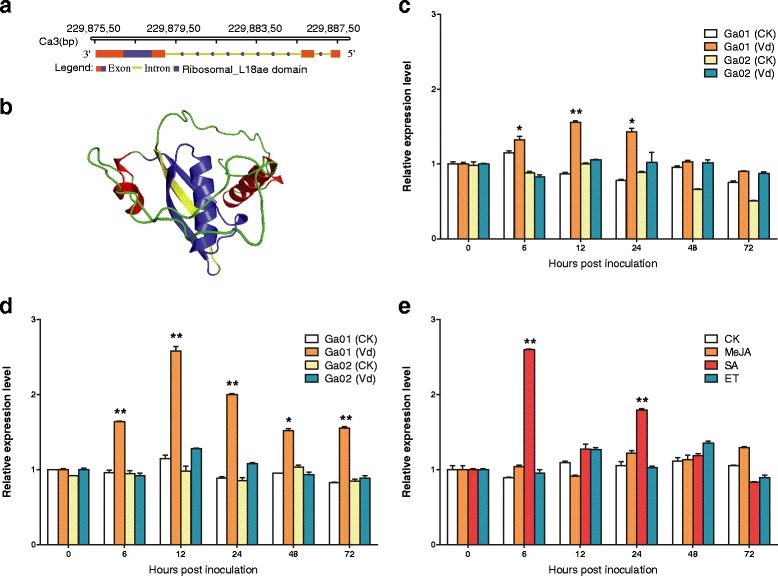
Structural analysis and expression patterns of *GaRPL18* under *Verticillium dahliae* stress or hormone treatment in cotton. **a** Gene structure analysis. **b** Protein 3D structure. **c,d** After seeding for 3 weeks, the cotton seedlings of *‘*HuNanChangDeTieZiMian’ (resistant, Ga01) and *‘*NaShangQuXiaoHua’ (susceptible, Ga02) were inoculated with *V. dahliae* spores or an equal sterile distilled water (Mock control). The sample of c is the leaf, while d is root. **e** The leaves of resistant cotton (*‘*HuNanChangDeTieZiMian’) were treated by exogenous hormones or an equal sterile distilled water. The Mock and treated roots and leaves were harvested at 0, 6, 12, 24, 48, and 72 hpi, and expression levels were determined by qRT-PCR using *Gahiston3* as the internal reference gene. Expression levels of Mock control was normalized to ‘1’, error bars represent the standard deviation of three biological replicates. Asterisks indicate statistically significant differences, as determined by Student’s *t*-test (*P <0.05; **P <0.001)

Based on the results of an unpublished transcriptomics analysis of disease responses in *G. arboreum* plants, we analyzed *GaRPL18* fragment sequences*.* ‘HuNanChangDeTieZiMian’ and ‘NaShangQuXiaoHua’ plants are resistant and susceptible to *Verticillium* wilt, respectively*.* We cloned the 1211-bp full-length *GaRPL18* sequence using the GaRPL18-F and GaRPL18-R primers (Table 1) along with ‘HuNanChangDeTieZiMian’ cDNA. *GaRPL18* was mapped to chromosome Ca3. It was localized to the complementary strand of the reference genome between positions 22987558 bp and 22988768 bp. The Gene Structure Display Server 2.0 program was used to analyze gene structures. Exons and introns are displayed as colored boxes and green lines, respectively (Fig. 1a). *GaRPL18* contains two introns as well as three exons that are 49, 64, and 450 bp long (from right to left). The gene consists of a 450-bp opening reading frame encoding 149 amino acids (Fig. 1a). *GaRPL18* also contains a conserved ribosomal_L18ae domain sequence (indicated with a blue box in Fig. 1a). The theoretical isoelectric point and molecular weight of the encoded protein were calculated as 10.46 and 17.9KDa, respectively, according to the ExPASy online tool. The 3D structure revealed that the ribosomal_L18ae domain (indicated in blue in Fig. 1b) is largely composed of beta turns and alpha helices. Because the GaRPL18 function is unknown, especially in *V. dahliae*-infected cotton plants, we examined *GaRPL18* expression patterns in samples harvested from *Verticillium* wilt-resistant and -susceptible cotton lines at different time points after inoculations*.* The *GaRPL18* expression levels were stable over the duration of the experiment in the control plants treated with water (Fig. 1c, d). In contrast, *GaRPL18* expression levels fluctuated in the susceptible cotton plants. Additionally, in the *Verticillium* wilt-resistant cotton plants treated with *V. dahliae*, *GaRPL18* expression was considerably upregulated at 6 h after inoculation, and peaked after 12 h, in both roots and leaves. To identify the signal pathway associated with *GaRPL18*, we examined the *GaRPL18* expression patterns in hormone-treated ‘HuNanChangDeTieZiMian’ plants. We observed that *GaRPL18* expression was differentially affected by the plant hormones. *GaRPL18* expression was rapidly induced, reaching a peak level at 6 h after treatment, following the application of exogenous SA, but remained at baseline levels following MeJA or ET treatments (Fig. 1e). These results suggest that GaRPL18 enhances the resistance of cotton to *V. dahliae*, and affects the SA-mediated signaling pathway.

### Interactions between *GaRPL18* and *PR* genes

**Fig. 2:**
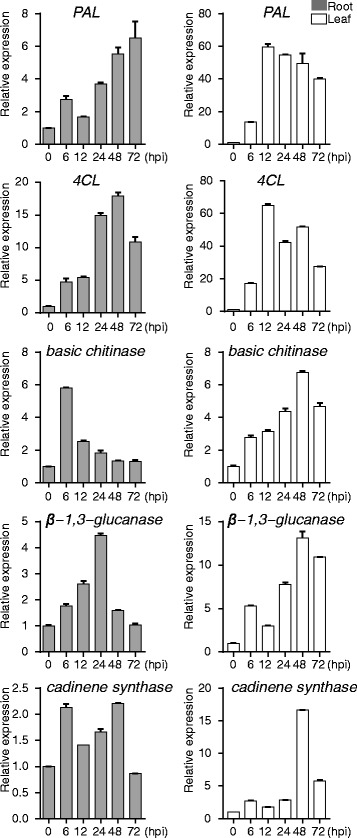
Interactions between *GaRPL18* and other *pathogenesis related* (PR) genes. Time course in the transcription of resistant cotton defense-related *pathogenesis related* (PR) genes from different time points after inoculation. Total RNAs were extracted from 3-week-old cotton plants after treatment with *V. dahliae*, and infected roots and leaves were harvested at 0, 6, 12, 24, 48, and 72 hpi. The expression levels of PR gene were determined by qRT-PCR using *Gahiston3* as the internal reference gene. Error bars represent the standard deviation of three biological replicates. Asterisks indicate statistically significant differences, as determined by Student’s *t*-test (*P <0.05; **P <0.001). The left part is the result of root (*gray*), the right part is the result of leaf (*white*)

To further clarify the effects of *GaRPL18* on *Verticillium* wilt resistance in cotton plants, we monitored the expression levels of *PR* genes at different time points after wilt-resistant cotton plants were inoculated with *V. dahliae*. Phenylpropanoid metabolism is critical for cotton defense responses, and involves the core genes *phenylalanine ammonia lyase* (*PAL*; Cotton_A_00465) and *4-coumarate: CoA ligase* (*4CL*; Cotton_A_02864) [51]. In leaf tissue, the expression levels of the *GaRPL18-*related *PAL* and *4CL* genes were highest at 12 h, while the expression of *4CL* in the roots peaked after 48 h. Additionally, *PAL* expression levels continued to increase in inoculated plants. The *basic chitinase* (Cotton_A_36866) and *β-1, 3-glucanase* (Cotton_A_36866) genes are important for plant disease resistance because the encoded enzymes can decompose the cell walls of pathogens, thereby protecting plants from disease [27]. In leaves, the expression levels of *GaRPL18-*related *basic chitinase* and *β-1,3-glucanase* genes peaked after 48 h. In contrast, the highest *basic chitinase* and *β-1,3-glucanase* expression levels in the roots were observed after 6 h and 24 h, respectively. *Cadinene synthase* (Cotton_A_34493) is a key gene involved in synthesizing terpenoids, which are critical phytoalexins in cotton [52]. The *cadinene synthase* expression level rapidly increased in the leaves and roots of inoculated plants, peaking after 48 h (Fig. 2).

### Silencing of *GaRPL18* in cotton considerably weakened resistance to *Verticillium dahliae*

**Fig. 3:**
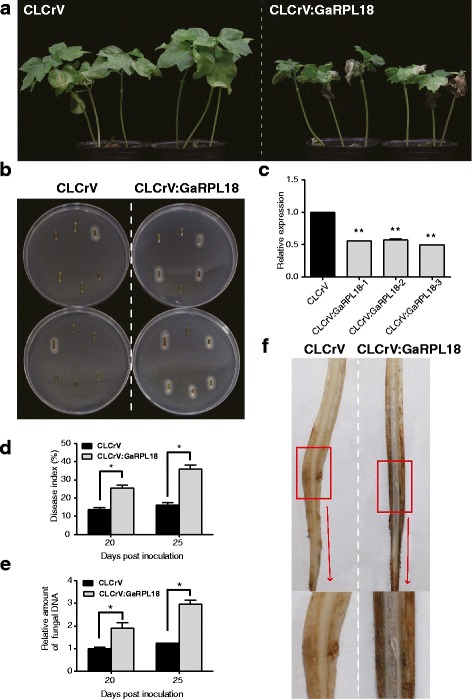
Ribosomal protein GaRPL18 is involved in the resistance of cotton to *V. dahliae*. **a** Disease symptoms induced with *V. dahliae* strain on the leaves of empty-vector control plants and CLCrV:GaRPL18 plants at 25 dpi. **b** 25-dpi stem sections were plated on PDA medium, incubating at 25 °C. Photos were taken at 7 day after plating. **c** Transcript levels of *GaRPL18* in leaves of empty-vector control plants and CLCrV:GaRPL18 plants, which were determined by qRT-PCR using *Gahiston3* as the internal reference gene. Value of CLCrV was normalized to ‘1’. **d** Assessment of Disease Index (DI) at 20 dpi and 25 dpi. Error bars represent the standard deviation of three biological replicates (*n* ≥ 30). **e** The levels of *Verticillium dahliae* biomass by qRT-PCR on the leaves of empty-vector control plants and CLCrV:GaRPL18 plants at 20 dpi and 25 dpi. Error bars represent the standard deviation of three biological replicates. Asterisks indicate statistically significant differences, as determined by Student’s *t*-test (*P <0.05; **P <0.001). **f** The infected CLCrV:GaRPL18 plants showed vascular browning in xylem at 25 dpi

To clarify the function of *GaRPL18* in cotton responses against *V. dahliae*, we used CLCrV-based VIGS to generate *GaRPL18-*knockdown plants*.* Two weeks after ‘HuNanChangDeTieZiMian’ plants underwent an *Agrobacterium tumefaciens* infiltration, we used qRT-PCR to assess the gene silencing efficiency. The abundance of *GaRPL18* transcripts was significantly lower in CLCrV:GaRPL18 plants than in the controls (Fig. 3c), indicating that *GaRPL18* was effectively silenced in these plants. Additionally, following *V. dahliae* inoculations, there were no obvious disease symptoms on the tissues of the vector control plants, while necrotic, yellowish, stunted, and wilting leaves were observed on CLCrV:GaRPL18 plants (Fig. 3a). Using a recovery assay, we examined the extent of the *V. dahliae* colonization in the infected stems of treated plants. This analysis was used to determine the virulence of the fungus for each plant. There were more fungal colonies in CLCrV:GaRPL18 plants than in CLCrV control plants (Fig. 3b). We used DI values to confirm that the resistance of cotton plants to *V. dahliae* was compromised in the absence of *GaRPL18* expression. The DI values were significantly higher for CLCrV:GaRPL18 plants than for the control plants at 20 and 25 days post-inoculation (dpi) (Fig. 3d).

**Fig. 4:**
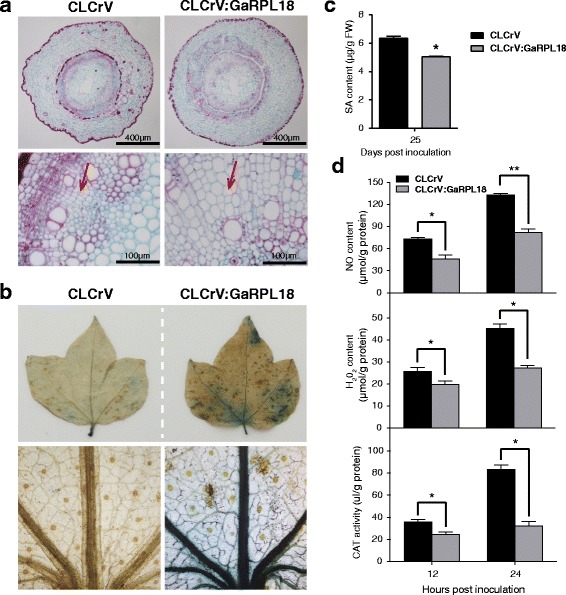
Effect of silencing of *GaRPL18* in cotton under *V. dahliae* stress. **a** The observation of cell morphology of the stems of CLCrV and CLCrV:GaRPL18 cotton plants at 25 dpi. **b** Trypan blue staining was used to visualize cell death of the leaves of CLCrV and CLCrV:GaRPL18 cotton plants at 25 dpi. **c** Measuring the SA content of CLCrV and CLCrV:GaRPL18 cotton plants at 25 dpi via HPLC. Bars represent standard error from three biological replicates. Asterisks indicate statistically significant differences (Student’s *t*-test; *P <0.05). **d** Leaves of CLCrV and CLCrV:GaRPL18 cotton plants were harvested at 12 hpi and 24 hpi to measure the contents of NO, H_2_O_2_ and CAT. Error bars represent the standard deviation of three biological replicates. Asterisks indicate statistically significant differences when compared with control (Student’s *t*-test; *P <0.05; **P <0.001)

Using qRT-PCR, we quantified *V. dahliae* colonization levels. The expression levels of *V. dahliae* genes indicated that CLCrV:GaRPL18 plants were significantly more sensitive to *V. dahliae* than the controls (Fig. 3e). Moreover, we observed that the xylem of CLCrV:GaRPL18 plants exhibited greater vascular browning than the controls (Fig. 3f). An additional examination of cotton cell morphology in inoculated plants revealed that the vascular bundle cells of CLCrV:GaRPL18 plants were longer and larger than those of control plants (Fig. 4a). This was likely because *V. dahliae* mycelia may have more easily penetrated CLCrV:GaRPL18 plant than the control. To visually contrast the differences in the leaves of plants inoculated with *V. dahliae*, we used trypan blue to stain dead cells. The trypan blue staining area was larger and the color was more intense in the CLCrV:GaRPL18 leaves, particularly in the veins, than in the control leaves (Fig. 4b).

### Salicylic acid, nitric oxide, H_2_O_2_, and catalase levels decreased in *GaRPL18*-silenced cotton plants upon *Verticillium dahliae* infection

To further evaluate the role of *GaRPL18* and its relationship with the SA pathway in cotton defense responses to *V. dahliae* infection, we analyzed the SA content and the abundance of several other immune-responsive compounds (i.e., NO, H_2_O_2_, and CAT) in *V. dahliae*-treated plants. All compounds were present in lower amounts in inoculated CLCrV:GaRPL18 plants than in CLCrV control plants (Fig. 4c, d). These results indicate that silencing *GaRPL18* in wilt-resistant cotton plants inhibits *V. dahliae*-induced production of SA, NO, H_2_O_2_, and CAT, further confirming that *GaRPL18* is closely related to the SA signaling pathway.

### Transgenic *Arabidopsis thaliana* seeds overexpressing *GaRPL18* increased in size and weight, while seedlings became more resistant to *Verticillium dahliae*

**Fig. 5:**
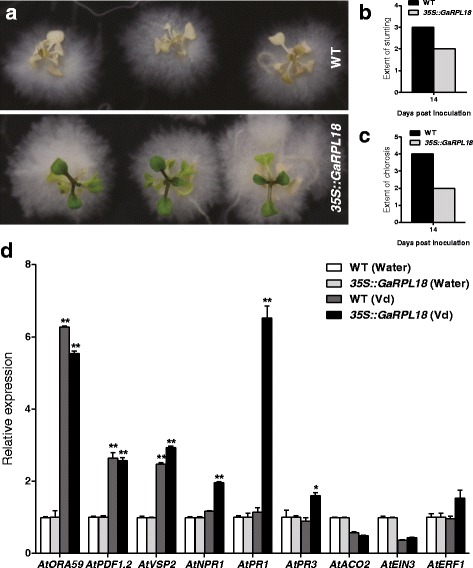
Effects of overexpression of *GaGRPL18* in *A. thaliana* seedings and the effects of signaling pathway-related marker genes under *V. dahliae* stress. **a** Disease symptoms of in vitro grown seedings. 2-week-old plantlets were treatment with *V. dahliae.* Photos were taken at 14 days after inoculated. **b** Extent of stunting in WT and *35S::GaRPL18* plants at 14 dpi. **c** Extent of leaf chlorosis in WT and *35S::GaRPL18* plants at 14 dpi. WT : wild-type. **d** Expression patterns of JA, SA and ET signaling pathway-related marker genes in WT and *GaRPL18* transgenic *A. thaliana* under water or *V. dahliae* challenge for 24 h. The expression levels was monitored by qRT-PCR using *AtUBQ10* as an internal control gene. Expression levels of untreated control was normalized to ‘1’. Error bars represent the standard deviation of three biological replicates. Asterisks indicate statistically significant differences, as determined by Student’s *t*-test (*P <0.05; **P <0.001). WT: wild-type

*Arabidopsis thaliana* ecotype Col-0 was transformed to generate transgenic lines overexpressing *GaRPL18* under the control of the 35S promoter. We then used a variety of methods to select homozygous *GaRPL18*-overexpressing transgenic lines, including a 0.1% Basta treatment, PCR, and qRT-PCR (Additional file 1: Figure S1a–e). We observed that the seeds of stable *GaRPL18*-overexpressing T_4_ plants were larger than those of WT plants (Additional file 1: Figure S1f). Analyses of seed weight per 1,000 mature dried seeds, seed length, seed width, and the length: width ratio revealed that the values were higher in *GaRPL18-*overexpressing transgenic plants than in WT controls, although this difference was not significant for the length: width ratio (Additional file 1: Figure S1g–i). We then used an in vitro technique to assess the resistance of *A. thaliana* seedlings to *V. dahliae*. The *GaRPL18*-overexpressing transgenic plants were compared with the WT plants daily. As expected, the transgenic *A. thaliana* plants were more resistant to *Verticillium* wilt than the WT plants. All WT plants were dead within 14 days of being inoculated with *V. dahliae*, while the transgenic plants were still alive (Fig. 5a). In WT plants, wilting was observed as early as 5 dpi, and the plants rapidly became yellow and exhibited stunted growth. At 14 dpi, the WT leaves were observed to be more stunted (grade 3) than the transgenic leaves (grade 2). Additionally, chlorosis was more severe in WT plants (grade 4) than in transgenic plants (grade 2) (Fig. 5b, c). This further confirms that the transgenic *A. thaliana* plants overexpressing *GaRPL18* were more resistant to *Verticillium* wilt than the WT plants.

### Effects of *GaRPL18* overexpression on the salicylic acid signaling pathway in *Verticillium dahliae*-treated transgenic *Arabidopsis thaliana* plants

We examined the expression level of signaling pathway-related genes, including those involved in JA biosynthesis [i.e., *octadecanoid-responsive* Arabidopsis *AP2/ERF domain protein 59* (*AtORA59*), *plant defensin 1.2* (*AtPDF1.2*), and *vegetative storage protein 2* (*AtVSP2*)], SA biosynthesis [i.e., *non-expressor of pathogenesis-related genes 1* (*AtNPR1*), *pathogenesis-related gene 1* (*AtPR1*), and *pathogenesis-related gene 3* (*AtPR3*)], and ET biosynthesis [i.e., *amino cyclopropane carboxylate oxidase 2* (*AtACO2*), *ethylene insensitive 3* (*AtEIN3*), and *ethylene response factor 1* (*AtERF1*)]. As shown in Fig. 5D, over-expression of *GaRPL18* in the Col-0 background did not affect expression of genes related to the JA, SA and ET pathways; these marker genes had similar expression levels in WT and over-expression plants without *V. dahliae* stress. In addition, the expression levels of the JA- and ET-related marker genes were similar overall between the WT controls and *GaRPL18-* overexpressing transgenic *A. thaliana* plants at 24 h post-inoculation (hpi). However, the SA-related genes were expressed more highly in the rosette leaves of transgenic plants than in the WT leaves at 24 h post-inoculation. The expression levels in the WT leaves were unchanged (Fig. 5d). Thus, *GaRPL18* overexpression had little effect on the signal transduction pathways regulating JA and ET biosynthesis, but had a significant effect on the SA biosynthesis signal transduction pathway.

### Application of exogenous salicylic acid increases the resistance of transgenic *Arabidopsis thaliana* plants to *Verticillium dahliae*

**Fig. 6:**
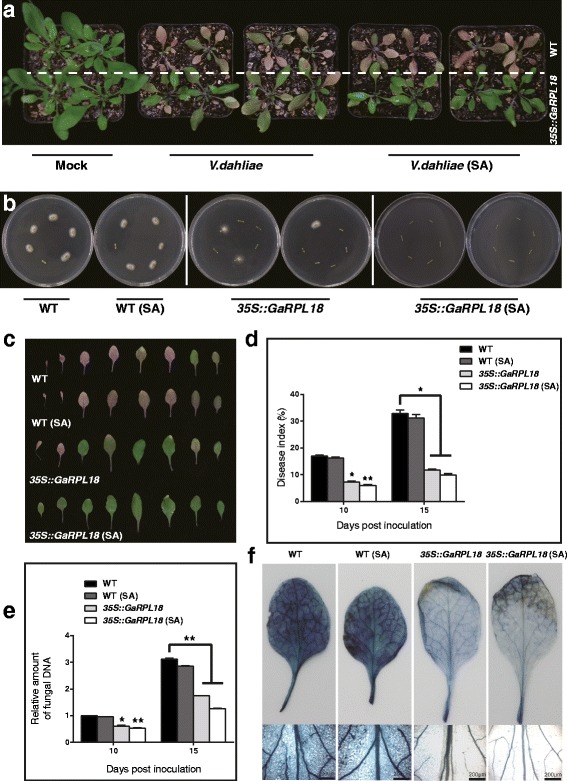
Effects of -overexpression of *GaRPL18* and exogenous SA in defense response to *V. dahliae* in *A. thaliana.* **a** Disease symptoms induced with *V. dahliae* strain on the rosette leaves of WT, *GaRPL18* overexpressed plants and exogenous SA-treated plants. 3-week-old seedings were inoculated with *V. dahliae,* and pretreated transgenic *A. thaliana* and wild-type seedlings rosette leaves with 1 mM SA for 24 h before inoculation with *V. dahliae*. Then, supplementing exogenous SA every day until four days. The control plants were sprayed with water. Photos were taken at 15 dpi. **b** 15-dpi stem sections were plated on PDA medium, incubating at 25 °C. Photographed at 7 day after plating. **c** The observation of *A. thaliana* rosette leaves. **d** Assessment of Disease Index (DI) at 10 dpi and 15 dpi. Error bars represent the standard deviation of three biological replicates (*n* ≥ 30). Asterisks indicate statistically significant differences when compared with control (Student’s *t*-test; *P <0.05; **P <0.001). **e** Quantification the biomass of *V. dahliae* by qRT-PCR using *AtUBQ10* as an internal control gene on the rosette leaves of WT, *GaRPL18* overexpressed plants and SA-treated plants at 10 dpi and 15dpi. Error bars represent the standard deviation of three biological replicates. Asterisks indicate statistically significant differences, as determined by Student’s *t*-test (*P <0.05; **P <0.001). **f** Trypan blue staining was used to visualize cell death of the leaves of *A. thaliana* at 15 dpi

We examined the resistance of *GaRPL18*-overexpressing transgenic *A. thaliana* plants to *V. dahliae* infection. When T_4_ lines were inoculated with *V. dahliae*, the transgenic plants were noticeably more resistant to the fungus than the WT plants. We then pretreated the rosette leaves of WT and transgenic *A. thaliana* seedlings with 1 mM SA for 24 h. The leaves were then inoculated with *V. dahliae* and treated daily with supplemental exogenous SA for 4 days. The transgenic *A. thaliana* plants were considerably more resistant to *V. dahliae* than the WT plants following the SA treatments (Fig. 6a). Additionally, we used the recovery assay to examine the colonization of infected WT and transgenic stems by *V. dahliae*. The number of colonies differed significantly between the WT, transgenic, and SA-treated transgenic plants. Overall, the transgenic plants grew better than the WT plants, with the SA-treated transgenic plants exhibiting the greatest resistance to *V. dahliae*. There were no differences between the WT and SA-treated WT plants (Fig. 6b). This is further evidence of the relationship between *GaRPL18* and SA. To more clearly observe the effects of *V. dahliae* on rosette leaves, we removed the rosette leaves from plants at 15 dpi. Consistent with our previous results, the SA treatment enhanced the resistance of transgenic *A. thaliana* rosette leaves to *V. dahliae*, but had little effect on WT leaves (Fig. 6c).

We calculated the DI values and quantified the *V. dahliae* colonization using qRT-PCR for WT plants, SA-treated WT plants, transgenic plants, and SA-treated transgenic plants. The DI value for WT plants was significantly higher than that of transgenic plants, including those treated with SA (Fig. 6d). Analyses of the expression level of fungal genes revealed that SA-treated transgenic plants were the most disease-resistant, while WT plants were the most susceptible (Fig. 6e). The trypan blue staining results were similar for WT and SA-treated WT plants. We observed dark blue veins and large stained mesophyll areas. The veins of transgenic leaves were light blue, and the mesophyll was not extensively stained. These observations are consistent with the greater disease resistance of SA-treated transgenic plants (Fig. 6f).

## Discussion

*Verticillium* wilt is one of the most lethal fungal diseases of plants and significantly reduces the quality and annual yields of cotton. Therefore, clarifying the molecular mechanism underlying cotton resistance to *V. dahliae* is essential for developing new wilt-resistant cotton varieties. Previous studies involving *V. dahliae* infections revealed that this pathogen attacks susceptible plants by germinating on the roots. It then employs various mechanisms to move through the root cortex into the plant vascular system, allowing for further colonization of the plant. When the fungus has completely infected the plant, it produces microsclerotial spores that remain stable and dormant in the soil until they are exposed to a new host [53]. To breed new cotton varieties resistant to *Verticillium* wilt, more research is required to identify resistance-related genes. Technological improvements have facilitated the mining and functional analysis of genes. There are some reports that describe the application of genes encoding proteins associated with resistance to *V. dahliae* to develop transgenic *A. thaliana* plants capable of inhibiting disease development. These genes include those encoding glutathione S-transferase [54, 55], rate-limiting enzymes [56], and transcriptional regulators [57]. However, efforts involving gene mining have been insufficient, and the cotton-producing regions of China are still affected by annual outbreaks of *Verticillium* wilt. The lack of known wilt-resistance genes in cotton makes the identification and functional characterization of *V. dahliae* resistance-related genes essential for the development of new wilt-resistant cotton varieties. In this study, we confirmed that *GaRPL18*, which encodes an RP, influences resistance to *Verticillium* wilt.

The function and characteristics of RPs during protein synthesis have been thoroughly researched, and recent in-depth studies confirmed that RPs also have important roles in other processes [20–22]. In particular, some studies have shown PRs play an important role in plant disease resistance [23, 24]. Recent cancer studies concluded that the RPs can regulate the expression of key genes in the MDM2–p53 regulatory loop, thereby promoting p53 activity and suppressing tumor growth [25, 26, 58–60]. Additionally, the RP has other effects related to disease resistance. For example, mutations to *RPS19* are associated with a congenital erythroblastopenia with a decreased abundance or lack of erythroid precursors [61]. However, little is known about the function and characteristics of RP-encoding genes during cotton resistance to *V. dahliae*. In this study, we determined that the RP mediates the resistance of cotton plants to *V. dahliae*.

To confirm the role of *GaRPL18* in cotton pathogen defense responses, we cloned the gene using cDNA produced from RNA extracted from wilt-resistant cotton plants. We also analyzed the *GaRPL18* expression patterns in different cotton varieties treated with *V. dahliae.* The *GaRPL18* expression level was rapidly and considerably upregulated in infected wilt-resistant cotton plants, but was relatively unchanged in the wilt-susceptible cotton plants. We then examined the expression of *PR* genes in wilt-resistant cotton plants. We confirmed that cotton resistance to *V. dahliae* results from interactions among various disease resistance systems that function together to resist pathogen invasions. Using VIGS technology, we determined that the silencing of *GaRPL18* in wilt-resistant cotton plants significantly decreased resistance to *V. dahliae*. Additionally, the NO, H_2_O_2_, and CAT contents were lower in cotton plants in which *GaRPL18* was silenced than in vector-control plants. An invasion by *V. dahliae* induces a spike in NO content, which is important for disease resistance in plants [62]. An earlier study concluded that the H_2_O_2_ content is significantly higher in disease-resistant tomato plants than in control plants, and that CAT plays an important role in early defense responses [63]. Our results indicate that NO, H_2_O_2_, and CAT affect the *GaRPL18*- related responses to *V. dahliae* in cotton plants. These observations confirm that *GaRPL18* is important for cotton resistance to *V. dahliae*, which is consistent with the anti-carcinogenic effects reported for other RPs. We used transgenic *A. thaliana* plants to further analyze *V. dahliae* disease resistance traits based on DI values, *V. dahliae* colonization, trypan blue staining, and recovery assays. Moreover, while the rosette leaves of WT plants gradually exhibited disease symptoms with increasing fungal growth and spread, almost no disease symptoms were observed on the rosette leaves of *GaRPL18-*overexpressing transgenic plants. This implies that the fungus was inhibited in the roots of the transgenic plants, and prevented from spreading into the rosette leaves. Additionally, in vitro analyses of the wilt-resistance of *GaRPL18-*overexpressing *A. thaliana* plants further confirmed the role of *GaRPL18* in plant defense responses to *V. dahliae*. This suggests that the accumulation of GaRPL18 considerably impedes pathogen colonization, even at the seedling stage. In summary, we confirmed that *GaRPL18-*overexpressing transgenic plants were more resistant to *V. dahliae* than WT plants, and that the extent of *V. dahliae* colonization was significantly lower in transgenic plants than in WT plants.

We also observed that *GaRPL18* expression was rapidly upregulated by SA, reaching peak levels 6 h after the application of the hormone. In contrast, MeJA and ET treatments had minimal effects on *GaRPL18* expression. Therefore, we hypothesized that *GaRPL18* may be associated with the SA signaling pathway. This was verified by a significant decrease in SA levels in *V. dahliae*-infected CLCrV:GaRPL18 plants. The activation of SA signaling in stressed plants can stimulate the expression of downstream disease-resistance genes to provide protection from pathogens [64, 65]. There are many genes related to the SA signaling pathway, including *NPR1*, *PR1*, *PR3*, *WRKYs*, and *AtGSTF6* [66–69]*.* To verify these results, the JA-, SA- and ET-related marker gene expression levels were examined in *V. dahliae*-infected transgenic *A. thaliana* plants overexpressing *GaRPL18*. The upregulated expression of SA signaling pathway-related genes (i.e., *AtNPR1*, *AtPR1*, and *AtPR3*) and SA content confirmed our hypothesis. To further clarify the link between *GaRPL18* and SA signaling, we treated the transgenic and WT plants with exogenous SA, and observed that the hormone significantly increased the resistance of transgenic plants to *V. dahliae*. These findings imply that GaRPL18, working in concert with the SA signaling pathway, has a strongly antimicrobial effect on *V. dahliae*. Therefore, *GaRPL18* may be useful for breeding *Verticillium* wilt-resistant cotton varieties.

## Conclusions

To the best of our knowledge, this study is the first to examine the RP function related to cotton resistance to *V. dahliae*. We used VIGS technology to confirm that *GaRPL18* is important for the resistance of cotton plants to *V. dahliae* infections. Our data also suggest that breeding new cultivars that overexpress *GaRPL18* may be an effective way to control *Verticillium* wilt of cotton plants. Finally, this study revealed that SA is an important factor related to the cotton defense response system, and that the mechanism of *GaRPL18*-associated *V. dahliae* resistance is related to the SA signaling pathway.

## Additional file

Additional file 1: Figure S1. Screening process of stable transgenic T4 lines and the effects of over-expressing *GaRPL18* in *A. thaliana.* a, b, c The process of product homozygous T_3_ lines. d Detection of positive lines. e The expression levels of *GaRPL18* were determined by qRT-PCR using *AtUBQ10* as an internal control gene. Error bars represent the standard deviation of three biological replicates. f The observation of seeds of *GaRPL18-*Transgenic *A. thaliana* and WT. g Seed weight per 1,000 mature dried seeds. h Seed length and seed width. i The ratio of length to width. (PDF 9473 kb)

## Abbreviations

4CL: 4-coumarate: CoA ligase
ACO2: Amino cyclopropane carboxylate oxidase 2
CAT: Catalase
CLCrV: *Cotton leaf crumple virus*
DI: Disease index
EIN3: Ethylene insensitive 3
ERF1: Ethylene response factor 1
ET: Ethylene
JA: Jasmonic acid
MDM2: Murine double minute 2
MeJA: Methyl jasmonate
NO: Nitric oxide
NPR1: Nonexpressor of pathogenesis-related gene 1
ORA59: Octadecanoid-responsive *Arabidopsis* AP2/ERF domain protein 59
PAL: Phenylalanine ammonia lyase
PDF1.2: Plant defensin 1.2
PR: Pathogenesis related
RP: Ribosomal protein
SA: Salicylic acid
VIGS: Virus-induced gene silencing
VSP2: Vegetative storage protein 2
WT: Wild-type
