# Supplementary figures and images for: Salicylic acid-related cotton (Gossypium arboreum) ribosomal protein GaRPL18 contributes to resistance to Verticillium dahliae
Source: BMC Plant Biol. 2017 Mar 3;17:59. doi: 10.1186/s12870-017-1007-5 (PMC5335750; doi:10.1186/s12870-017-1007-5)

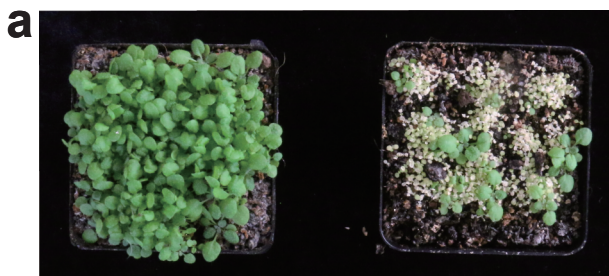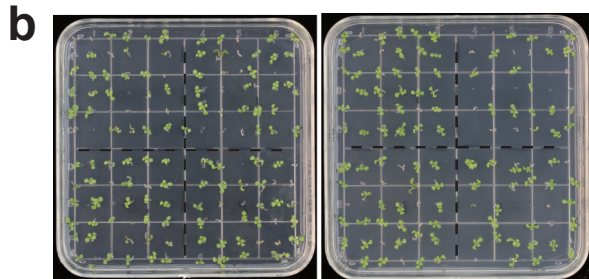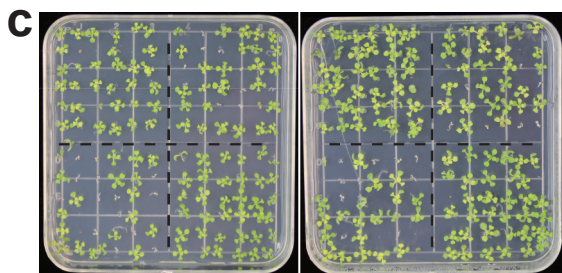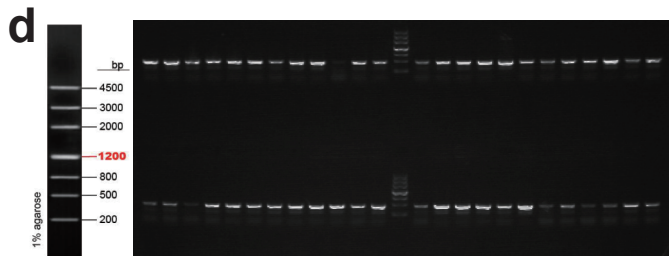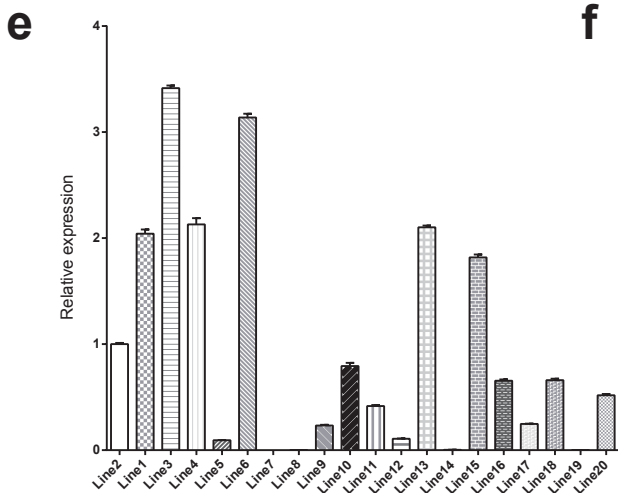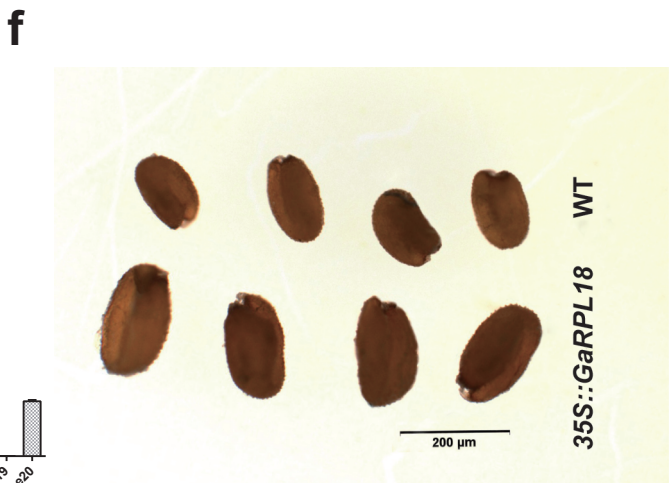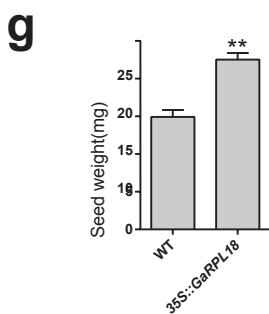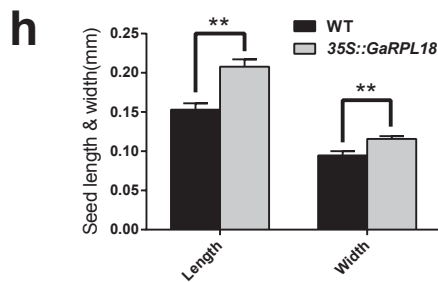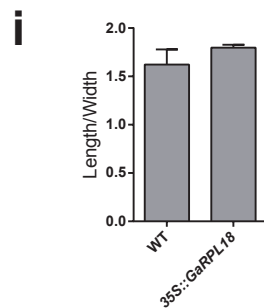

Supplement: Additional file 1: Figure S1. — Screening process of stable transgenic T4 lines and the effects of over-expressing GaRPL18 in A. thaliana. a, b, c The process of product homozygous T3 lines. d Detection of positive lines. e The expression levels of GaRPL18 were determined by qRT-PCR using AtUBQ10 as an internal control gene. Error bars represent the standard deviation of three biological replicates. f The observation of seeds of GaRPL18-Transgenic A. thaliana and WT. g Seed weight per 1,000 mature dried seeds. h Seed length and seed width. i The ratio of length to width. (PDF 9473 kb) [file 12870_2017_1007_MOESM1_ESM.pdf]
